# To Chemo or Not to Chemo… That Is the Question

**DOI:** 10.1200/GO.23.00164

**Published:** 2023-08-03

**Authors:** Maria Felisa Eva B. Martin, Frederic Ivan L. Ting

**Affiliations:** ^1^ Department of Medicine, Dr Pablo O. Torre Memorial Hospital, Bacolod City, Philippines; ^2^ Department of Clinical Sciences, College of Medicine, University of St La Salle, Bacolod City, Philippines

## Abstract

The best practices and clinical consensus statements often differ from real-world practice.

Mary Jane is a hard-working 34-year-old mother who has spent most of her time raising her four children and taking care of the household chores in the small Filipino town of Calatrava. Despite living in poverty, she and her family had everything they thought they needed: food, water, and a home in which to rest at the end of each day.

Mary Jane's children encouraged her to seek medical help when she started losing a significant amount of weight and began having episodes of stabbing abdominal pain. Mary Jane did not want to be a burden to her family, and their stability was shaken at the thought of their mother being seriously ill. After weeks of trying to withstand the pain, she reluctantly agreed to visit a local clinic. The doctors examined her, performed basic laboratory tests (ie, complete blood count, electrolytes, urinalysis, and fecal analysis), and sent her home with medications for gastritis. Despite taking these medications as prescribed, Mary Jane's abdominal pain persisted. Within 6 weeks, she developed noticeable jaundice and excruciating back pain, extending down her right leg, leaving her unable to walk. Finally, after 8 weeks, she presented to the nearby hospital and was admitted for further workup.

The nearby hospital was a rural community hospital with multiple limitations, from very limited blood examinations to lack of IV contrast. During her admission, imaging revealed a calcified mass in her pancreas, with liver nodules, lung lesions, and lymph nodes that appeared to be metastatic. Tumor markers were drawn, but had to be sent to the nearest island, which was a 2-hour boat ride away. Her CA 19-9 level returned after 7 days and was clearly elevated. Although the constellation of symptoms and data were concerning for metastatic pancreatic cancer, Mary Jane was discharged with analgesics and a referral to a medical oncologist, who recommended a biopsy. Undergoing a tissue biopsy would mean traveling for hours to a private hospital in the main city and would likely cost more than Mary Jane and her family could afford. Mary Jane refused the recommendation for biopsy to save the few remaining pesos she had for possible cancer treatment.

This scenario is not uncommon to medical oncologists in developing countries, especially in the rural areas. Empathetic physicians are often tempted to offer empiric chemotherapy versus delay management while patients scramble to find the money and other resources they need to proceed with a more thorough workup.

Unlike first-world countries, cancer care in developing countries is unique because more often than not, access to the most basic diagnostic tests and treatment facilities is limited.^1,2^ Long and relatively expensive travels, the struggle of ensuring that the family has food to eat because of the missed opportunity to receive their daily wage, and the concentration of subspecialists in the major cities are some of the several barriers that need to be hurdled before being able to receive proper treatment from an oncologist in the Philippines. Thus, almost all types of cancers tend to be more extensive with an advanced stage at initial consult, subsequently compounding the burden of treatment.^3^ Hence, the dilemma as to whether empiric chemotherapy should be given leaves the physicians and patients at a crossroad—having to grapple with the accepted standard of care versus the quick and pragmatic option to decide which action would be more beneficial.

Although Mary Jane's clinical symptoms and initial diagnostic test results may point toward a possible malignancy, a diagnosis of cancer requires confirmation since infectious causes such as disseminated tuberculosis are endemic in the Philippines. Thus, her medical oncologist was very hesitant to offer empiric chemotherapy and strongly advised a biopsy. Mary Jane would have wanted to follow the necessary procedures advised by her physician, but she was very hesitant because of financial constraints. Fortunately, as her pain persistently worsened, a distant relative reached out and offered financial help.

She was finally admitted to a tertiary hospital in the capital city, which was 4 hours away from her residence, for the diagnostic workup and treatment. She was referred to a radiation oncologist, who offered the option of undergoing palliative radiotherapy for the relief of her back and leg pain should her lesions prove to be malignant. The plan gave Mary Jane hope for a pain-free and comfortable future albeit recognizing the likelihood of a terminal disease. Surprisingly, a computed tomography scan with contrast revealed circular or ovoid lesions with peripheral enhancement characteristic of disseminated tuberculosis with pulmonary, nodal, and hepatic involvement; nonetheless, malignancy still could not be ruled out. A biopsy was finally performed.

With the clinical picture, the available diagnostic results, and the knowledge of the endemicity of tuberculosis in the Philippines, Mary Jane's medical oncologist considered an infectious cause of her signs and symptoms, rather than cancer. Definitive treatment was put on hold until the histology results were finalized. Ultimately, her biopsy results were inconclusive—showing neither malignant cells nor granuloma, which is seen in tuberculosis. Mary Jane's team of physicians decided to commit to the diagnosis of disseminated tuberculosis as this condition is common in the Philippines and may present with multiple lymphadenopathies similar to a malignancy. She was started on treatment for tuberculosis and subsequently showed marked improvement during regular follow-ups after she was discharged. She has gained back the weight that she had lost. The pain in her abdomen, back, and right leg has disappeared. Most importantly, she has been able to return home and resume her roles of wife and mother.

There are many patients in developing countries, especially in the Philippines, who share stories that are similar to Mary Jane's. In her case, her doctors were fortunately able to arrive at the right diagnosis after a multidisciplinary team discussion and prevent unnecessary complications of treatment from chemotherapy. However, most people who live in resource-poor settings continue living their entire lives without seeing a doctor, or if they do, it is already too late.^4,5^ Although ideas and plans have been laid out, the Philippine health care system has not yet been able to fully serve the marginalized in society.^1,2^

Physicians in developing countries inadvertently learn to accommodate their patients' wishes to minimize expenses to spare their families from further spiraling down into poverty. Since most clinical trials and researches are performed in affluent countries, there are limited studies on the different host and environmental factors that are uniquely found in low- and middle-income countries, such as malnutrition or tuberculosis that may affect treatment tolerance, failure, or success.^6,7^

The dilemma as to whether to treat with empirical chemotherapy deeply bothers and oftentimes pushes medical oncologists against the wall, especially those working in rural settings. Mary Jane's story epitomizes the fact that the best practices, clinical consensus statements, and practice guidelines often differ from real-world practice choices and decisions.
